# Supplementary material for: Hierarchized phosphotarget binding by the seven human 14-3-3 isoforms
Source: Nat Commun. 2021 Mar 15;12:1677. doi: 10.1038/s41467-021-21908-8 (PMC7961048; doi:10.1038/s41467-021-21908-8)
Supplement: Supplementary file 1 — Supplementary information [file 41467_2021_21908_MOESM1_ESM.pdf]

SUPPLEMENTARY INFORMATION

Hierarchized phosphotarget binding by the seven human 14-3-3 isoforms

Gogl et al.

Contents:

Supplementary table 1. Structures of the 14-3-3/motif III peptide complexes available in the PDB
Supplementary figure 1. Classification of 31 PBM-containing HPV-E6 proteins
Supplementary figure 2. Competitive FP measurements with the entire family of human 14-3-3 proteins
Supplementary figure 3. Production of the 14-3-3ζ chimera with the 18E6 phosphopeptide
Supplementary figure 4. The arrangement of the chimera molecules in the crystal structures
Supplementary figure 5. Molecular interface between 14-3-3ζ and phospho-18E6 PBM in the absence or presence of FSC
Supplementary figure 6. Water-mediated contacts
Supplementary figure 7. Analysis of 14-3-3/phosphotarget complexomes
Supplementary figure 8. Sequence divergence trend for the seven human 14-3-3 isoforms
Supplementary figure 9. Additional data on 14-3-3/phosphotarget complexomes
Supplementary References

Supplementary table 1. Structures of the 14-3-3/motif III peptide complexes available in the PDB. FSC – fusicoccin, cotA – cotylenin A, "modified" indicates that the peptide sequence or length are not authentic. Red bold font highlights the peptides matching the pS/pT**XX**-COOH motif III consensus.

PDB ID	Motif III peptide	(pS/pT)Xn-COOH, n=	14-3-3 partner	Complex contents	Resolution	Ref.
**1O9D**	..QSY**pT**V-COOH	1	PMA2	1433 + peptide	**2.30 Å**	1
**1O9F**	..QSY**pT**V-COOH	1	PMA2	1433 + peptide +FSC	**2.70 Å**	1
**3E6Y**	..QSY**pT**V-COOH	1	PMA2	1433 + peptide +cotA	**2.50 Å**	2
**5NWI**	..YFS**pS**N-COOH	1	KAT1	1433 + peptide	**2.35 Å**	3
**5NWJ**	..YFS**pS**N-COOH	1	KAT1	1433 + peptide	**2.07 Å**	3
**5NWK**	..YFS**pS**N-COOH	1	KAT1	1433 + peptide +FSC	**3.30 Å**	3
**3IQU**	.QRST**pS**T-COOH	1	Raf1 (modified)	1433 + peptide	**1.05 Å**	4
**3IQV**	.QRST**pS**T-COOH	1	Raf1 (modified)	1433 + peptide +FSC	**1.20 Å**	4
**3P1N**	.KRRK**pS**V-COOH	1	TASK-3	1433 + peptide	**1.40 Å**	5
**3P1O**	.KRRK**pS**V-COOH	1	TASK-3	1433 + peptide +FSC	**1.90 Å**	5
**3P1P**	.KRRK**pS**V-COOH	1	TASK-3	1433 mutant + peptide	**1.95 Å**	5
**3P1Q**	.KRRK**pS**V-COOH	1	TASK-3	1433 mutant + peptide +FSC	**1.70 Å**	5
**3P1R**	.KRRK**pS**V-COOH	1	TASK-3	1433 mutant + peptide	**1.70 Å**	5
**3P1S**	.KRRK**pS**V-COOH	1	TASK-3	1433 mutant + peptide +FSC	**1.65 Å**	5
**3SMK**	.KRRK**pS**V-COOH	1	TASK-3	1433 mutant + peptide +cotA	**2.10 Å**	5
**3SML**	.KRRK**pS**V-COOH	1	TASK-3	1433 mutant + peptide +FSC_deriv.	**1.90 Å**	5
**3SMN**	.KRRK**pS**V-COOH	1	TASK-3	1433 mutant + peptide +FSC_deriv.	**2.00 Å**	5
**3SP5**	.KRRK**pS**V-COOH	1	TASK-3	1433 mutant + peptide +cotA_deriv.	**1.80 Å**	5
**3SPR**	.KRRK**pS**V-COOH	1	TASK-3	1433 mutant + peptide +FSC_deriv.	**1.99 Å**	5
**3UX0**	.KRRK**pS**V-COOH	1	TASK-3	1433 mutant + peptide +FSC_deriv.	**1.75 Å**	5
**6GHP**	.KRRK**pS**V-COOH	1	TASK-3	1433 mutant + FSC_deriv.	**1.95 Å**	6
**3SMM**	.KRRK**pS**V-COOH	1	TASK-3	1433 mutant + FSC_deriv.	**2.00 Å**	5
**3SMO**	.KRRK**pS**V-COOH	1	TASK-3	1433 mutant + FSC_deriv.	**1.80 Å**	5
**4FR3**	.KRRK**pS**V-COOH	1	TASK-3	1433 mutant + FSC_deriv.	**1.90 Å**	5
**4JC3**	.GFPA**pT**V-COOH	1	ERα	1433 + peptide	**2.05 Å**	7
**4JDD**	.GFPA**pT**V-COOH	1	ERα	1433 + peptide +FSC	**2.10 Å**	7
**5N10**	.GFPA**pT**V-COOH	1	ERα	1433 + peptide +ring stabilizer	**1.60 Å**	8
**6HHP**	.GFPA**pT**V-COOH	1	ERα	1433 + peptide +stabilizer 1	**1.80 Å**	9
**6HMT**	.GFPA**pT**V-COOH	1	ERα	1433 + peptide +stabilizer 2	**1.10 Å**	9
**6HKB**	.GFPA**pT**V-COOH	1	ERα	1433 + peptide +stabilizer 3	**1.70 Å**	9
**6HKF**	.GFPA**pT**V-COOH	1	ERα	1433 + peptide +stabilizer 4	**1.80 Å**	9
**6HN2**	.GFPA**pT**V-COOH	1	ERα	1433 + peptide +stabilizer 5	**1.70 Å**	9
**6HMU**	.GFPA**pT**V-COOH	1	ERα	1433 + peptide +stabilizer 6	**1.20 Å**	9
**6W0L**	SMRRM**pS**M-COOH	1	Henipah virus protein W	1433 + peptide	**2.30 Å**	10
**4O46**	**RTARpSKV-COOH**	**2**	**Influenza virus NS1**	**1433 + peptide**	**2.90 Å**	11
**6TWZ**	**RTRREpTQL-COOH**	**2**	**HPV16 E6**	**1433 + peptide**	**2.80 Å**	12
**6T80**	LRRN**pS**GCG-COOH	3	AANAT (modified)	1433σ-peptide chimera	**2.99 Å**	13

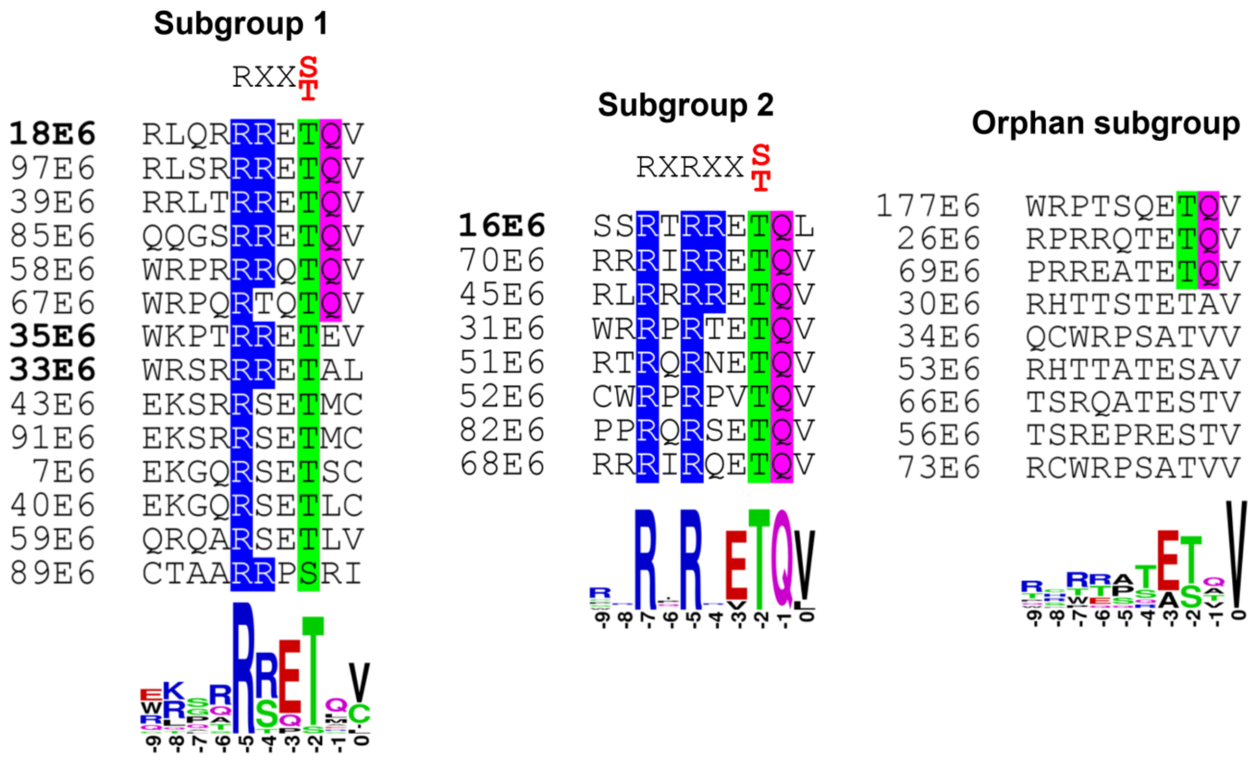

Supplementary figure 1. **Classification of 31 PBM-containing HPV-E6 proteins.** Classification is done based on the correspondence of their C-terminal PBMs to the consensus motifs phosphorylatable by basophilic kinases (subgroups 1 and 2). The third, orphan subgroup comprises PBMs of HPV-E6 proteins whose phosphorylation is less certain. Bold font marks the E6 PBMs used in this work. Note that many PBMs overlap with recognition motifs for phosphorylation by DNA damage response kinases ATM/ATR (TQ and SQ sites highlighted). Below are shown Weblogo diagrams [14] for the PBMs within each of the three subgroups (positions are numbered according to the PBM convention).

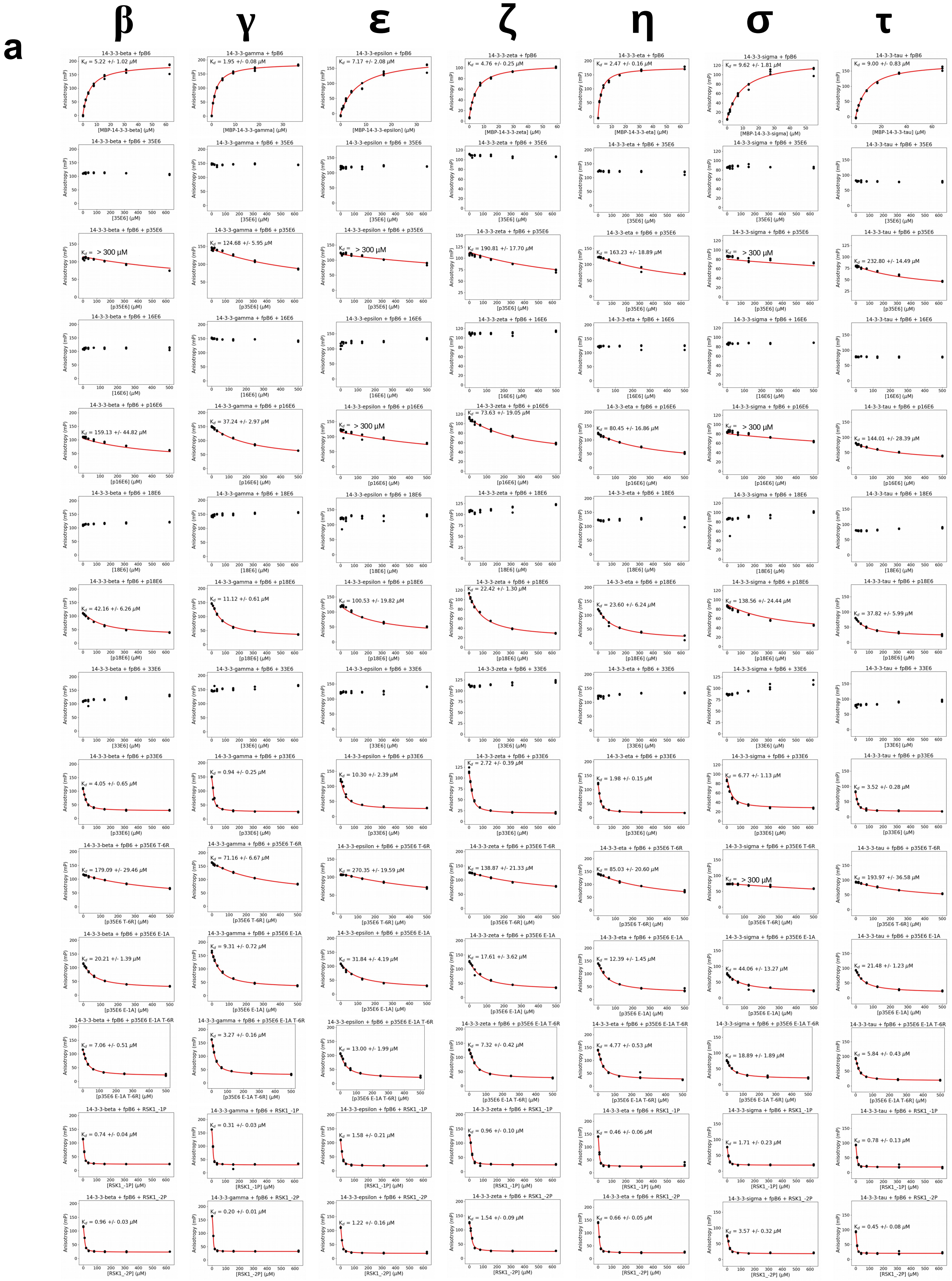

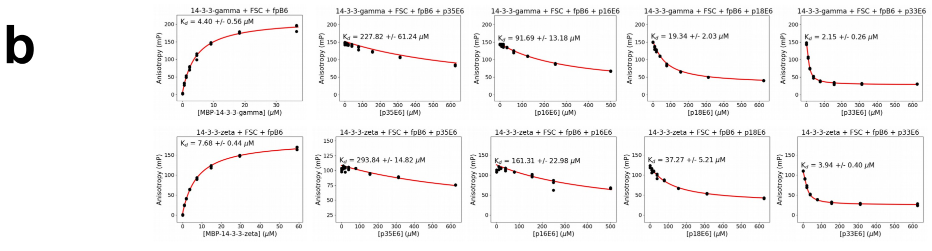

Supplementary Fig. 2
See the legend on the next page

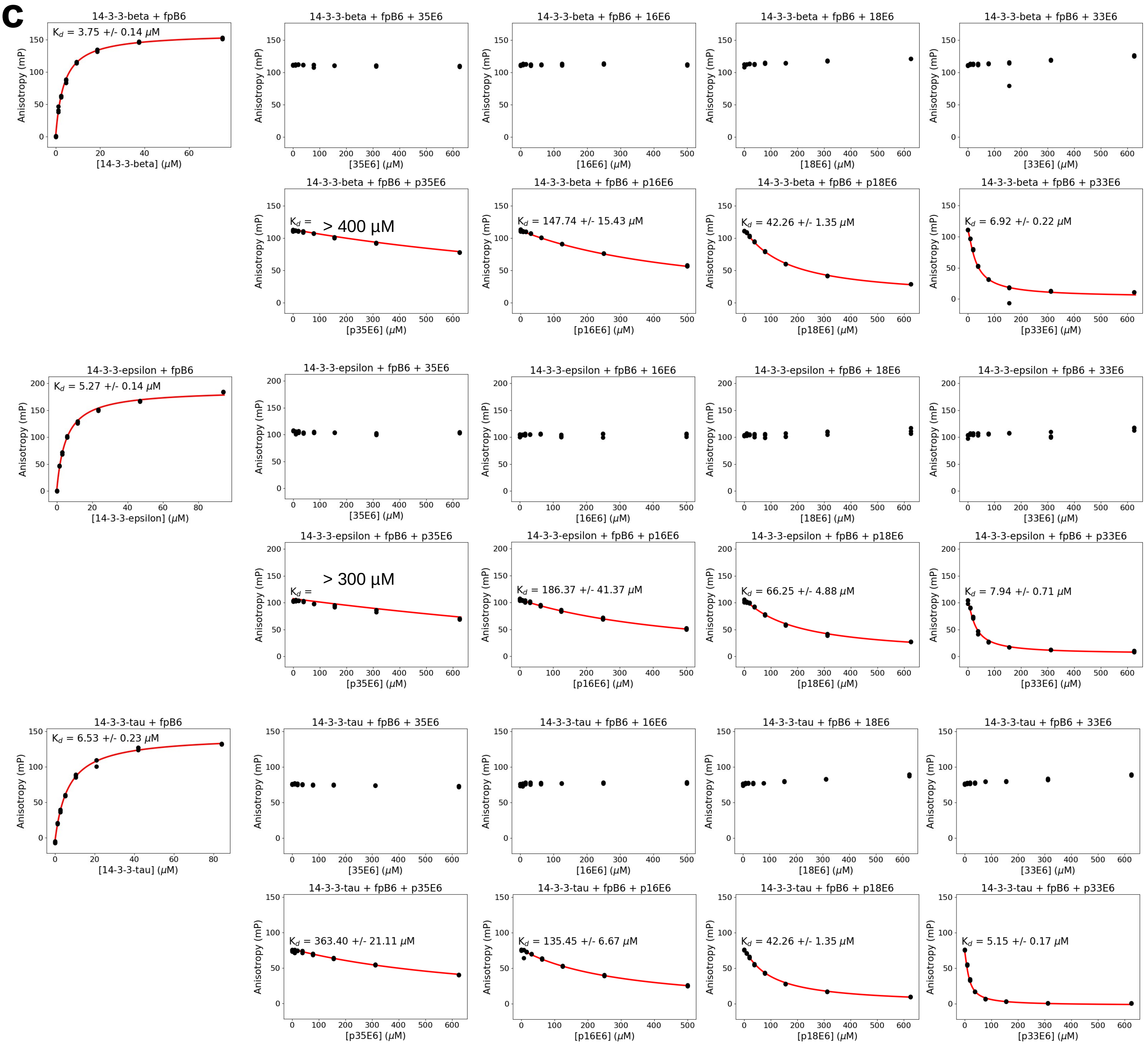

Supplementary figure 2. Competitive FP measurements with the entire family of human 14-3-3 proteins. a In each section, the first panel shows the direct FP experiments between the labeled peptide tracer and the titrated 14-3-3 protein and the following panels show competitive titrations. Competitive experiments were performed at a relatively high protein concentration to achieve 80% complex formation with the peptide tracer. Obtained polarization values were fitted with ProFit [15]. During competitive fitting, an experimental window close to the window of the direct experiment was either achieved without restraints or a restrained fitting was used. No fitted curve is shown if we did not observe a quantifiable competition. **b** Direct and competitive FP experiments in the presence of FSC. In **a** and **b** the full-length MBP-tagged human 14-3-3 isoforms were used. **c** FP measurements of affinities of three selected untagged 14-3-3 isoforms (beta, epsilon and tau) devoid of the flexible C-terminal tails against the four HPV E6 PBMs. Three graphs on the left correspond to the direct titrations of the three 14-3-3 constructs by the reporter peptide, other graphs correspond to the competitive titrations using HPV E6 PBMs. Unfitted 1, 3 and 5 rows correspond to the unphosphorylated HPV E6 PBMs (no binding); 2, 4 and 6 rows correspond to the phosphorylated HPV E6 PBMs (fitted using the ProFit [15] algorithm).

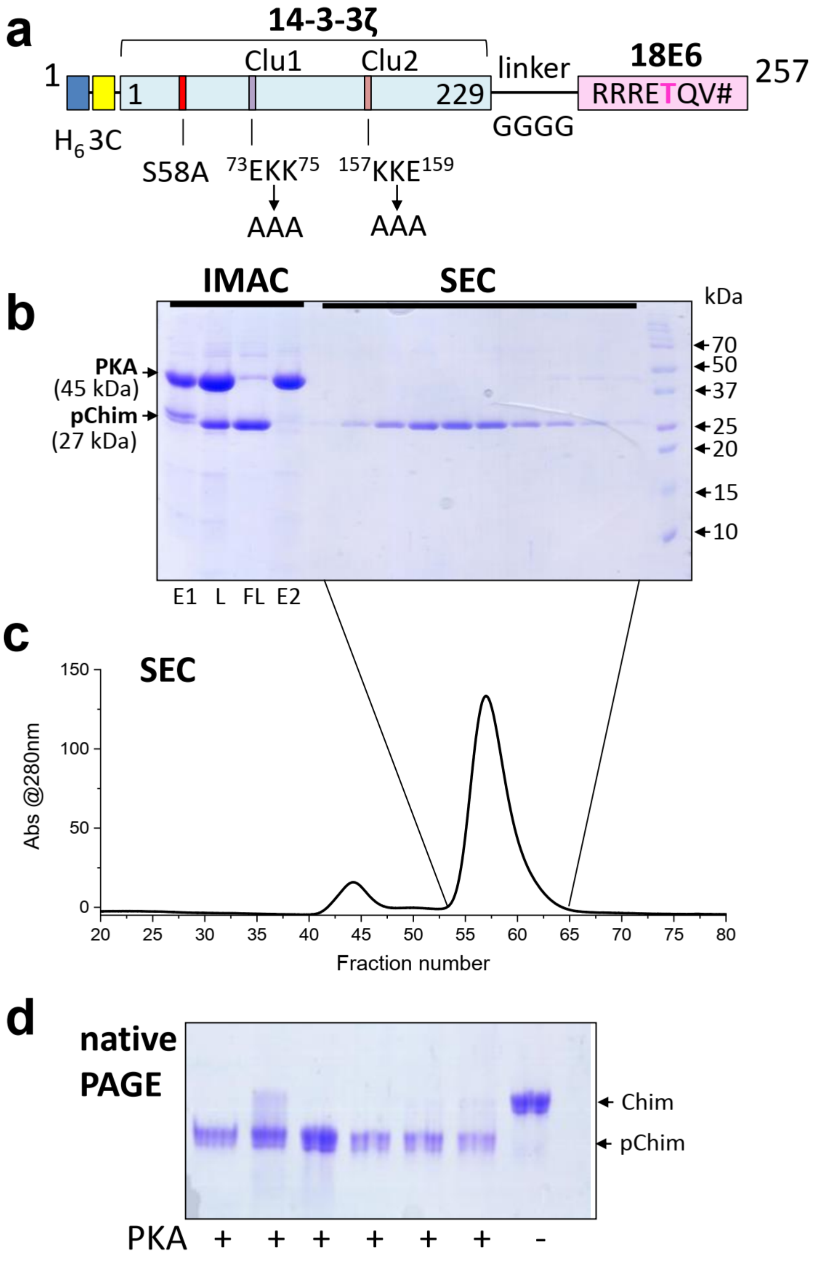

Supplementary figure 3. Production of the 14-3-3ζ chimera with the 18E6 phosphopeptide. a Schematic representation of the primary structure of the chimera. The His-tag (H6), 3C protease cleavage site (3C), 14-3-3ζ core modified to prevent phosphorylation of Ser58 and to promote crystallization by the surface entropy reducing mutations [16, 17] (highest scoring clusters 1 and 2 (clu1 and clu2, respectively) are marked), the GGGG linker and the 18E6 phosphorylatable peptide are indicated. The C-terminal carboxylic group of the 18E6 protein is denoted by #. **b** Purification of the chimera co-expressed with the His-tagged PKA by subtractive immobilized metal affinity and size-exclusion chromatography (IMAC and SEC) analyzed by SDS-PAGE (uncropped gel is shown): E1 – fraction bound on the HisTrap HP column, L – E1 fraction treated by 3C and reloaded on the HisTrap HP column, FL – unbound fraction containing the phosphorylated chimera, E2 – His-tagged PKA rebound on the column. Positions of PKA and chimera and their Mw values are shown by arrows. The data are representative of three independent protein purifications. **c** SEC profile of the chimera on a Superdex 75 26/60 column (GE Healthcare). **d** Native PAGE analysis of the purified chimera expressed in *E. coli* in the presence (+) or absence of PKA (-). Uncropped gel with six independently obtained batches of the phosphorylated chimera is shown. Note the higher electrophoretic mobility of the phosphorylated chimera due to additional negative charges conferred by phosphate moiety.

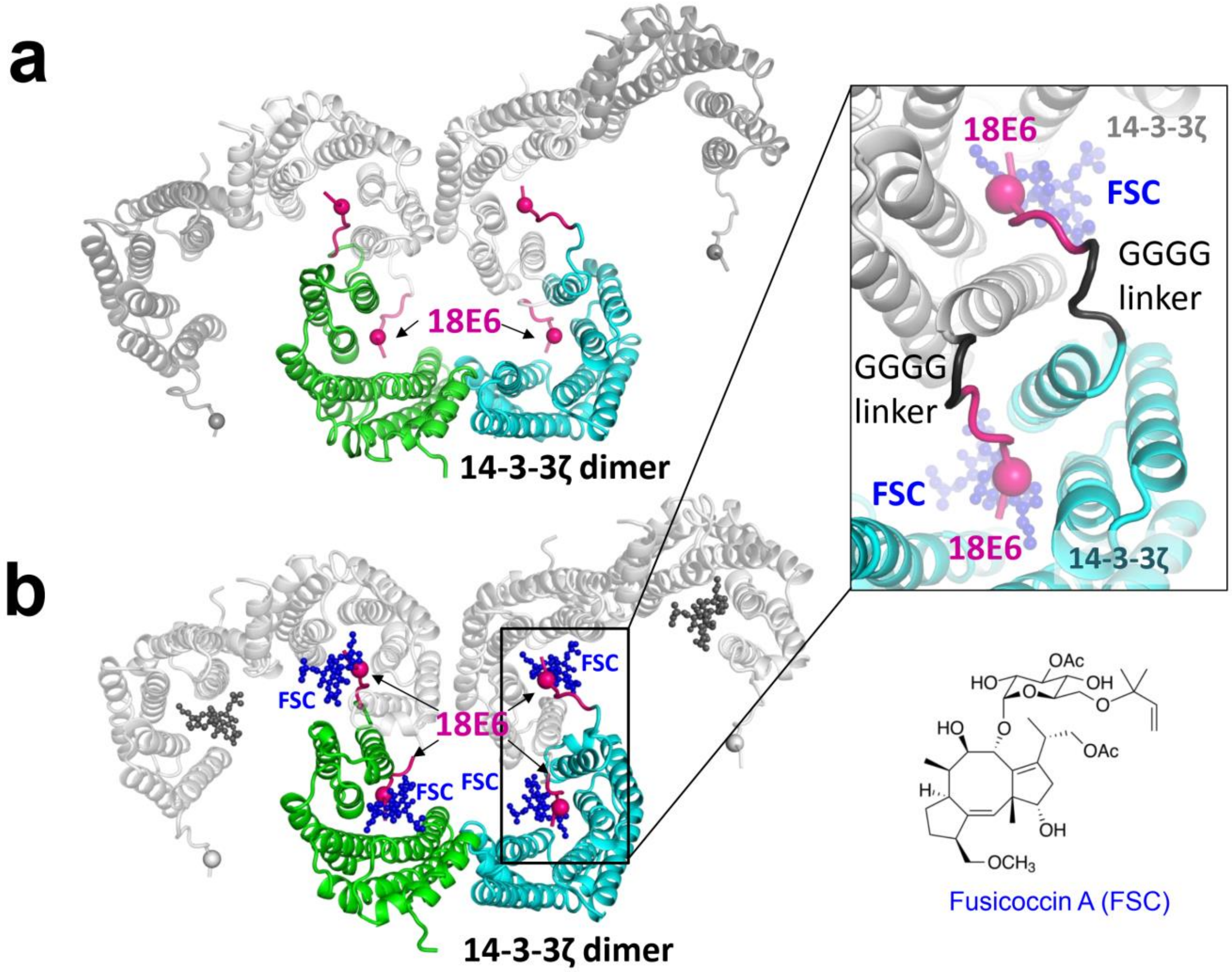

Supplementary figure 4. The arrangement of the chimera molecules in the crystal structures. The arrangement obtained in the absence (**a**) or presence of FSC (**b**) is shown. One 14-3-3ζ dimer of the asymmetric unit is colored, its crystallographically symmetric dimers are light grey. 18E6 phosphopeptides (magenta) are indicated by arrows, phospho-Thr residues are shown by spheres. The interdimer phosphopeptide swap stabilizing the supramolecular assembly is shown in a magnified view only for the FSC bound structure. A chemical formula of FSC is shown in the bottom-right corner.

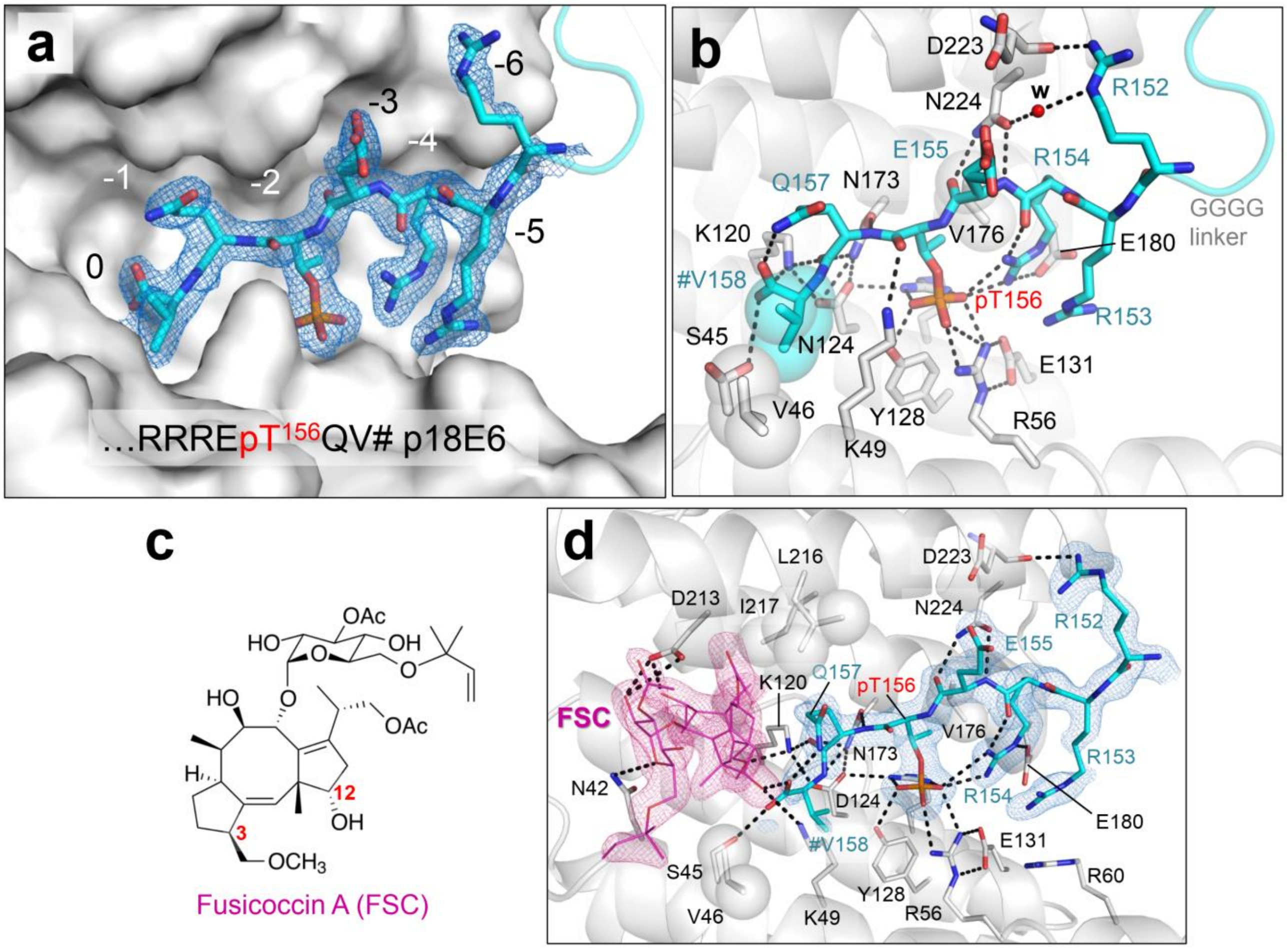

Supplementary figure 5. Molecular interface between 14-3-3ζ and phospho-18E6 PBM in the absence or presence of FSC. a A magnified view on one of the amphipathic grooves of 14-3-3ζ showing the 18E6 phosphopeptide conformation in the absence of FSC. The corresponding $2F_o$-F_c electron density maps contoured at 1σ are shown. Positions are numbered according to the PBM convention. **b** Polar contacts (dashed lines) and hydrophobic interactions (semitransparent spheres) stabilizing the bound 18E6 peptide conformation in the absence of FSC. **c** Chemical formula of FSC showing positions of the functional groups discussed in the text. **d** A closeup view on the ternary complex 14-3-3ζ/18E6 PBM/FSC showing polar contacts (dashed lines) and hydrophobic interactions (semitransparent spheres) positioning the 18E6 phosphopeptide (cyan sticks) and FSC (thin pink sticks) in the amphipathic groove of a 14-3-3ζ subunit (semitransparent light grey ribbon). $2F_o$-F_c electron density maps contoured at 1σ are shown for the peptide and FSC. # denotes the C-terminus (-COOH). The GGGG linker is omitted for clarity.

FSC binds in its well-defined cavity by hydrophobic interactions with Phe117, Ile166, Ile217 and Leu216, polar contacts with residues Asn42 and Asp213, and a remarkable H-bond involving its 3-metoxy oxygen and the side chain of Lys120 of 14-3-3ζ. The latter contact breaks the Lys120 interaction with the carboxyl-group of the 18E6 PBM formed in the absence of FSC, displacing the carboxyl to another position, where it establishes a new contact with the 12-hydroxy group of FSC. 14-3-3ζ Lys49 also switches its position, and loses a contact to the backbone carbonyl of pThr156 to establish instead a contact with the 12-hydroxy group of FSC. The side chain of the C-terminal Val158 of the 18E6 PBM shifts 3.5 Å towards the phosphate moiety of Thr156, breaking the hydrophobic contact with Val46 of 14-3-3ζ and significantly dispersing the local electron density. As a result, while most of the peptide conformation remained unchanged, the B-factors of the last 18E6 PBM residue in the refined FSC-bound structure increased significantly.

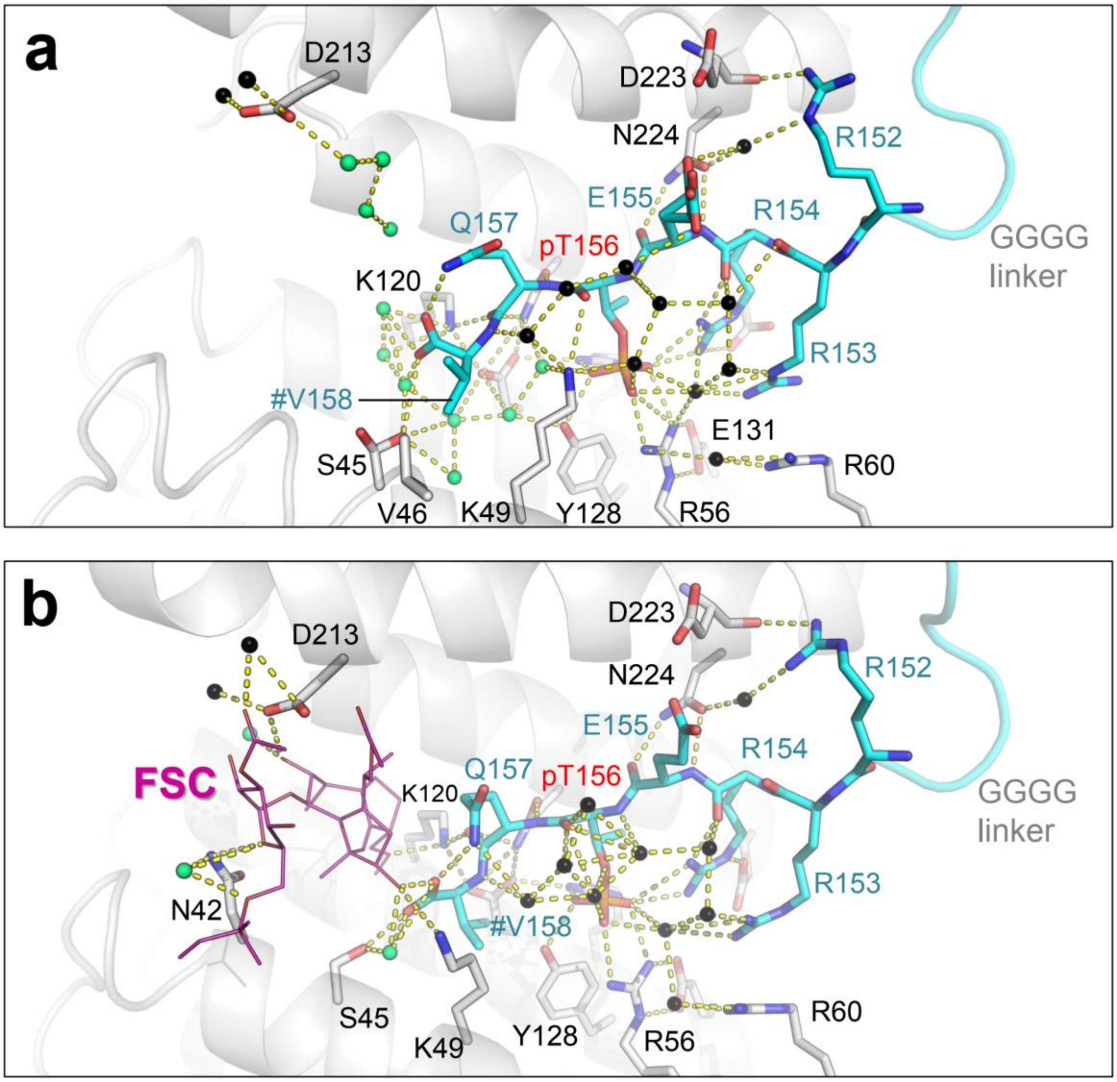

Supplementary figure 6. Water-mediated contacts. Comparison of the water-mediated polar contacts formed in the 14-3-3ζ/18E6 interface in the absence (**a**) or in the presence of fusicoccin (FSC) (**b**). The main residues involved in the interactions are shown by sticks with color coding: 14-3-3 residues are shown in light grey, 18E6 residues are in cyan, phospho-group of Thr156 is shown by orange sticks. FSC is shown by thin magenta sticks, water molecules affected by FSC binding are shown by lime green, those similar in two structures are black. The C-terminal 18E6 residue (V158) is denoted by #. Note the significant redistribution of water molecules upon FSC binding.

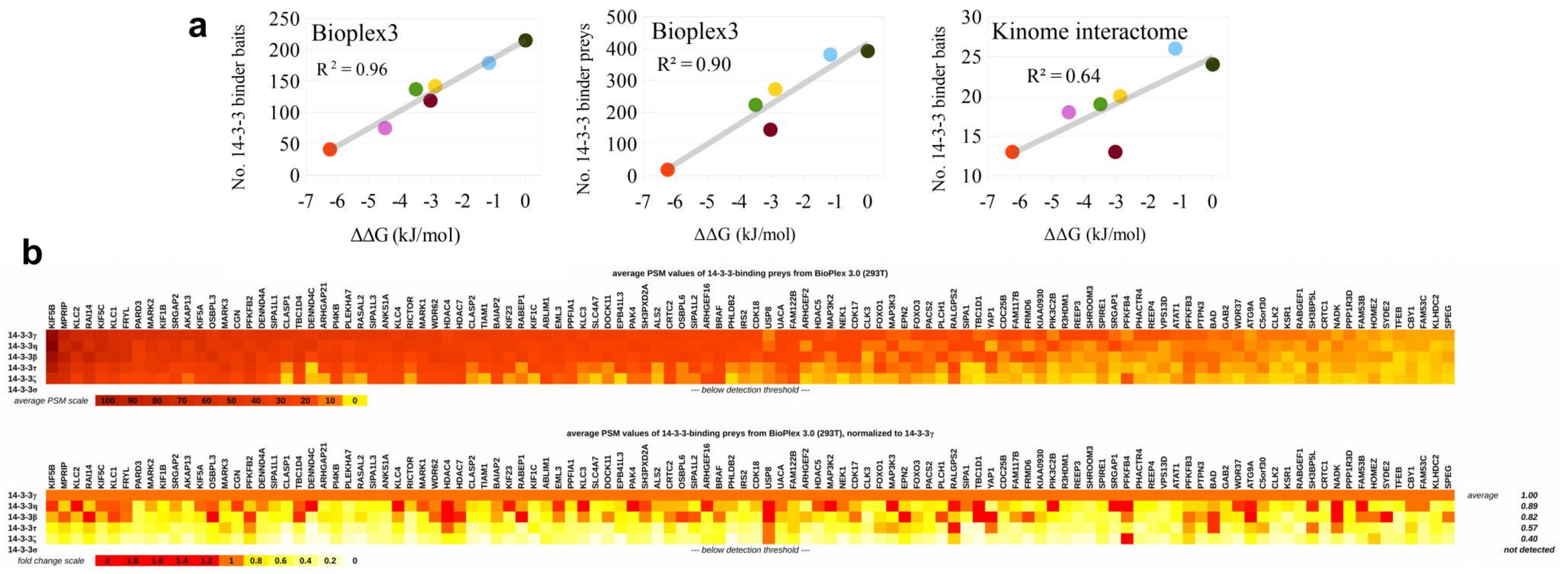

Supplementary figure 7. Analysis of 14-3-3/phosphotarget complexomes. a Correlation between the number of 14-3-3 interaction partners and the average free binding energy difference ΔΔG between the strongest phosphopeptide-binder, 14-3-3γ, and all individual isoforms (same color code as in Fig. 4d). $\Delta\Delta G$ values were calculated from the average K_D ratios from Fig. 4c. Interaction partners can be either "preys" or "baits" in the AP-MS experiment. Using the Bioplex database (https://bioplex.hms.harvard.edu and [18]), the number of baits are shown in the left panel and the number of preys are in the middle panel. The numbers of 14-3-3 binding baits are shown in the right panel using the kinome interactome as taken from (https://sec-explorer.shinyapps.io/Kinome_interactions/ and [19]). **b** A heatmap of *PSM* values of preys, detected with 5 common bait 14-3-3 proteins (upper part). Since 14-3-3ε was not included in the Bioplex database as a bait, the analysis was only performed for the rest isoforms. Upon normalization to the *PSM* value of the 14-3-3γ experiment (lower part), the relative *PSM* values of the 114 studied prey proteins roughly follow the same order of affinity that we define in this study (see Fig. 4). Although 14-3-3σ was included as a bait in the Bioplex database, it did not recognize any of these prey proteins above the detection threshold. Source data are provided as a Source Data file.

a

identity level

	removed:	C	-α1-α3-9,C	-α1α2/ΔC	-α1-α3	-α1-3/α9	-α1-3/α9-8	-α1-3/α9-7	-α1-3/α9-6	-α1-3/α9-5	-α1-5/α9-6
	left:	α1-α9	α1α2	α3-α9	α4-α9	α4-α8	α4-α7	α4-α6	α4α5	α4	α6
	**FULL**	**aa1-234**	**aa1-32**	**aa33-234**	**aa71-234**	**aa71-204**	**aa71-185**	**aa71-164**	**aa71-137**	**aa71-116**	**aa139-164**
**gamma**	100.0%	100.0%	100.0%	100.0%	100.0%	100.0%	100.0%	100.0%	100.0%	100.0%	100.0%
**eta**	87.0%	87.6%	84.8%	88.2%	86.0%	82.8%	81.7%	78.7%	83.6%	76.1%	65.4%
**beta**	73.2%	75.7%	73.5%	75.9%	73.5%	68.2%	67.3%	60.9%	67.7%	57.1%	46.2%
**zeta**	73.1%	76.0%	75.0%	75.9%	74.1%	69.7%	69.0%	63.0%	69.2%	59.5%	42.3%
**tau**	67.9%	71.7%	65.6%	72.4%	70.4%	63.6%	61.9%	54.3%	61.5%	47.6%	38.5%
**sigma**	63.6%	66.5%	62.5%	67.0%	65.2%	60.4%	60.0%	54.2%	59.4%	47.8%	35.7%
**epsilon**	60.3%	64.5%	60.6%	64.5%	62.8%	56.0%	53.9%	46.8%	50.7%	38.6%	34.6%
	1	2	3	4	5	6	7	8	9	10	11

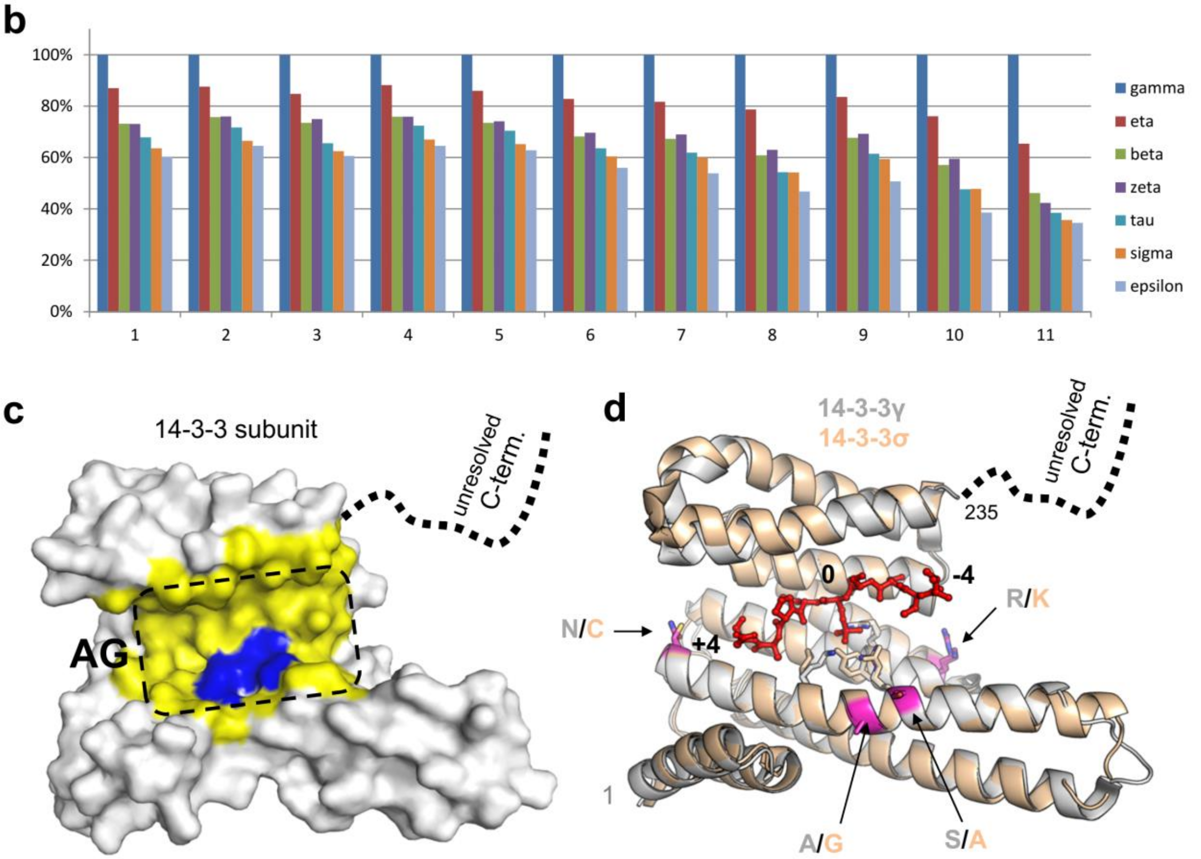

Supplementary figure 8. Sequence divergence trend for the seven human 14-3-3 isoforms. The trend is preserved throughout the entire 14-3-3 sequence, also for its different sub-regions (indicated from 1 to 11, starting from the full-length sequences). **a** The identity level relative to 14-3-3γ (the strongest binder) is shown in % and color-coded using the standard red-yellow-white scale for different human 14-3-3 isoforms, considering the full-length sequences (1) or different parts thereof (2-11). **b** The same identity levels as in **a** shown by histograms for clarity. Note that exclusion of the most variable flexible C-terminal peptides from analysis does not re-shuffle the positions of the 14-3-3 isoforms in the trend significantly. This indicates that the general target affinity differences arise from fine conformational effects spanning the entire structure, rather than a defined sub-region. **c** The phosphopeptide-binding amphipathic groove (yellow) and phosphate-coordinating pocket (blue) are identical in the seven human 14-3-3 isoforms (shown mapped on the surface of 14-3-3σ subunit). **d** Spatial overlay of the 14-3-3γ (6A5S) [20] and 14-3-3σ (5LU2) [21] structures with the bound phosphopeptide (red sticks), showing the four amino acid differences (magenta) located most close to, yet far enough from the phosphopeptide-binding groove.

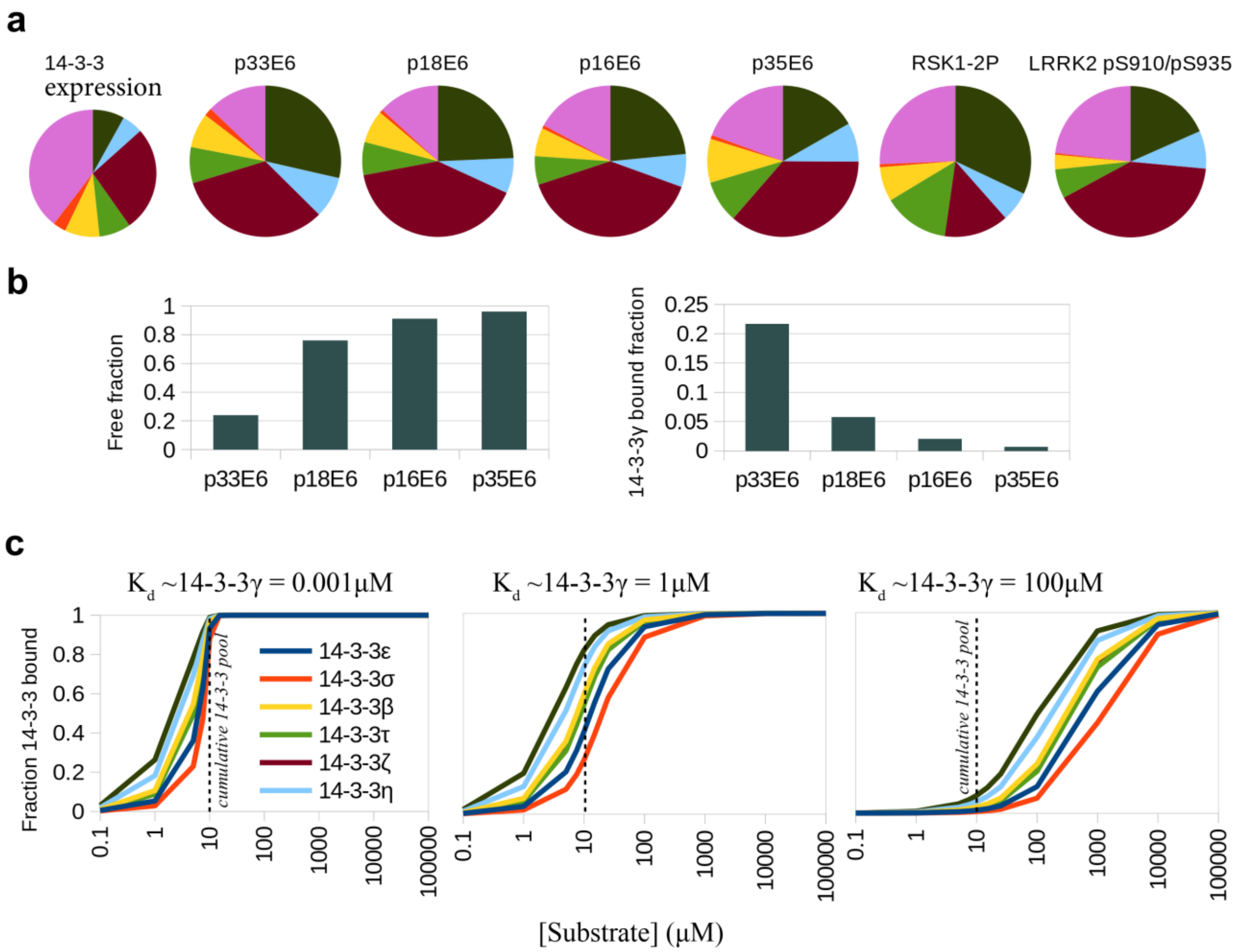

Supplementary figure 9. Additional data on 14-3-3/phosphotarget complexomes (Fig. 5). a Predicted proportions of 14-3-3-bound phosphoproteins that would be engaged with each individual isoform are mostly dependent on the proteomic context (see Fig. 5) and less dependent on the absolute affinity of the interaction partner. Predictions were performed using the experimental affinity values, assumed low target concentration (25 nM), and the proteomic context of uterus. Concentrations were calculated from abundancies from the PAXdb (https://pax-db.org and [22]), according to conversion rules described in the Methods section. **b** The amount of complex formation is strongly dependent on the absolute affinity of the interaction partner. The same prediction is shown as in **b**, but the amount of free target, or the formed complex with 14-3-3γ, are shown for the four studied E6 proteins. **c** By varying the concentration and affinity of a 14-3-3 target, it is possible to estimate how much target can sequester and sink the cellular 14-3-3 pool. In the case of a strong binder, an equimolar amount of target is sufficient to saturate the cellular 14-3-3 proteins. However, if the target affinity is relatively weak (>1 μM), the required target concentration can exceed the cumulative 14-3-3 concentration by orders of magnitudes. Source data are provided as a Source Data file.

Supplementary References

1. Wurtele M, Jelich-Ottmann C, Wittinghofer A, Oecking C. Structural view of a fungal toxin acting on a 14-3-3 regulatory complex. *EMBO J* **22**, 987-994 (2003).
2. Ottmann C, *et al.* A structural rationale for selective stabilization of anti-tumor interactions of 14-3-3 proteins by cotylenin A. *J Mol Biol* **386**, 913-919 (2009).
3. Saponaro A, *et al.* Fusicoccin Activates KAT1 Channels by Stabilizing Their Interaction with 14-3-3 Proteins. *Plant Cell* **29**, 2570-2580 (2017).
4. Molzan M, *et al.* Impaired binding of 14-3-3 to C-RAF in Noonan syndrome suggests new approaches in diseases with increased Ras signaling. *Mol Cell Biol* **30**, 4698-4711 (2010).
5. Anders C, *et al.* A semisynthetic fusicoccane stabilizes a protein-protein interaction and enhances the expression of K+ channels at the cell surface. *Chem Biol* **20**, 583-593 (2013).
6. Andrei SA, *et al.* Rationally Designed Semisynthetic Natural Product Analogues for Stabilization of 14-3-3 Protein-Protein Interactions. *Angew Chem Int Ed Engl* **57**, 13470-13474 (2018).
7. De Vries-van Leeuwen IJ, *et al.* Interaction of 14-3-3 proteins with the estrogen receptor alpha F domain provides a drug target interface. *Proc Natl Acad Sci U S A* **110**, 8894-8899 (2013).
8. de Vink PJ, Briels JM, Schrader T, Milroy LG, Brunsveld L, Ottmann C. A Binary Bivalent Supramolecular Assembly Platform Based on Cucurbit[8]uril and Dimeric Adapter Protein 14-3-3. *Angew Chem Int Ed Engl* **56**, 8998-9002 (2017).
9. Sijbesma E, *et al.* Site-Directed Fragment-Based Screening for the Discovery of Protein-Protein Interaction Stabilizers. *J Am Chem Soc* **141**, 3524-3531 (2019).
10. Edwards MR, *et al.* Henipavirus W Proteins Interact with 14-3-3 To Modulate Host Gene Expression. *J Virol* **94**, (2020).
11. Qin S, *et al.* Structural basis for histone mimicry and hijacking of host proteins by influenza virus protein NS1. *Nat Commun* **5**, 3952 (2014).
12. Gogl G, *et al.* Dual Specificity PDZ- and 14-3-3-Binding Motifs: A Structural and Interactomics Study. *Structure* **28**, 747-759 e743 (2020).
13. Sluchanko NN, Tugaeva KV, Titterington J, Antson AA. Unpublished work (2020).
14. Crooks GE, Hon G, Chandonia JM, Brenner SE. WebLogo: a sequence logo generator. *Genome research* **14**, 1188-1190 (2004).
15. Simon MA, *et al.* High-throughput competitive fluorescence polarization assay reveals functional redundancy in the S100 protein family. *FEBS J* **287**, 2834-2846 (2020).
16. Goldschmidt L, Cooper DR, Derewenda ZS, Eisenberg D. Toward rational protein crystallization: A Web server for the design of crystallizable protein variants. *Protein Sci* **16**, 1569-1576 (2007).
17. Goldschmidt L, Cooper DR, Derewenda ZS, Eisenberg D. SERp Server. (ed^(eds) (2007).
18. Huttlin EL, *et al.* Dual Proteome-scale Networks Reveal Cell-specific Remodeling of the Human Interactome. *bioRxiv*, 2020.2001.2019.905109 (2020).
19. Buljan M, *et al.* Kinase Interaction Network Expands Functional and Disease Roles of Human Kinases. *Molecular Cell* **79**, 504-520.e509 (2020).
20. Xu Y, Ren J, He X, Chen H, Wei T, Feng W. YWHA/14-3-3 proteins recognize phosphorylated TFEB by a noncanonical mode for controlling TFEB cytoplasmic localization. *Autophagy* **15**, 1017-1030 (2019).
21. Sluchanko NN, *et al.* Structural Basis for the Interaction of a Human Small Heat Shock Protein with the 14-3-3 Universal Signaling Regulator. *Structure* **25**, 305-316 (2017).
22. Wang M, Herrmann CJ, Simonovic M, Szklarczyk D, von Mering C. Version 4.0 of PaxDb: Protein abundance data, integrated across model organisms, tissues, and cell-lines. *Proteomics* **15**, 3163-3168 (2015).
