# Supplementary material for: Hierarchized phosphotarget binding by the seven human 14-3-3 isoforms
Source: Nat Commun. 2021 Mar 15;12:1677. doi: 10.1038/s41467-021-21908-8 (PMC7961048; doi:10.1038/s41467-021-21908-8)
Supplement: Supplementary file 3 — Description of Additional Supplementary Files [file 41467_2021_21908_MOESM3_ESM.docx]

File Name: Supplementary data file 1.zip

Description: A compressed file with the raw data of the FP measurements. Each subfolder contains the experimental data that were measured on a particular 14-3-3 protein. "dC" indicates C-terminally truncated 14-3-3 proteins, "FSC" indicates that the FP experiment was measured in the presence of fusicoccin. All files are in the format required by ProFit.

File Name: Supplementary Data 2.xls
Description: Numerical 14-3-3 complex formation calculator. The calculator numerically estimates the approximate amounts of complexes formed between a target protein and seven competing 14-3-3 proteins, by taking into account the total concentrations of each molecule and their individual experimentally determined (or assumed) dissociation constants.
